# Bounds on the distribution of the number of gaps when circles and lines are covered by fragments: Theory and practical application to genomic and metagenomic projects

**DOI:** 10.1186/1471-2105-8-70

**Published:** 2007-03-02

**Authors:** John Moriarty, Julian R Marchesi, Anthony Metcalfe

**Affiliations:** 1 Department of Mathematics/Boole Centre for Research in Informatics, University College Cork, Cork, Ireland; 2 Alimentary Pharmabiotic Centre, University College Cork, Cork, Ireland; 3 Department of Microbiology, University College Cork, Cork, Ireland

## Abstract

**Background:**

The question of how a circle or line segment becomes covered when random arcs are marked off has arisen repeatedly in bioinformatics. The number of uncovered gaps is of particular interest. Approximate distributions for the number of gaps have been given in the literature, one motivation being ease of computation. Error bounds for these approximate distributions have not been given.

**Results:**

We give bounds on the probability distribution of the number of gaps when a circle is covered by fragments of fixed size. The absolute error in the approximation is typically on the order of 0.1% at 10× coverage depth. The method can be applied to coverage problems on the interval, including edge effects, and applications are given to metagenomic libraries and shotgun sequencing.

## Background

The question of how a circle becomes covered when random arcs are marked off has arisen repeatedly in bioinformatics. As an example, a prokaryotic chromosome is typically circular and the clones extracted from it for genomic libraries or shotgun sequencing projects are randomly positioned arcs. The number of uncovered gaps is of particular interest: a genomic library ideally has no gaps, while one might seek to stop the undirected part of a shotgun sequencing project when a small number of gaps remain (we call this the 'stopping problem'). Coverage problems also arise in the culture-independent methods of metagenomics, since the number of clones coming from each genome in a mixed community is random. Accordingly, the question of the number of gaps has been treated by many authors, in both mathematical and biological contexts.

We refer the reader to [1] for a review of the mathematical literature on circle covering problems and the exact distribution of the number of gaps when all arcs are of equal length. Driven by practical considerations, approximate distributions for the number of gaps have been given in the genomics literature: see for example [2-4]. These approximate distributions are easier to compute than the exact distributions. Some address modified coverage problems with particular biological relevance, such as the 'edge effects' which arise when certain arc positions cannot occur. Bounds for the probability of completely covering the circle are given in [5], but to the knowledge of the authors no bounds have been given for the distribution of the number of gaps. In this paper we give bounds for the probability distribution of the number of gaps in circle covering problems. The method can be applied to coverage problems on the interval, including edge effects. Applications are given to metagenomic libraries and the stopping problem in shotgun sequencing.

## Results

**Proposition 1 ***Suppose that n arcs, each of length s, are placed uniformly and independently at random on a circle of circumference 1. Then the number of gaps has approximately the Poisson distribution with parameter m *= *n*(1 - *s*)^*n*-1^. *The error in the approximation is given in Proposition 2*.

**Proposition 2 ***In the setting of Proposition 1, let W denote the number of gaps and let Y denote a Poisson random variable with parameter m. Then for any nonnegative integer w*,

|*P*(*W *≤ *w*) - *P*(*Y *≤ *w*)| ≤ *ε*

where

ε=n2(1−s)2(n−1)−n(n−1)(1−2s)+n−1max⁡(1,m)
 MathType@MTEF@5@5@+=feaafiart1ev1aaatCvAUfKttLearuWrP9MDH5MBPbIqV92AaeXatLxBI9gBaebbnrfifHhDYfgasaacH8akY=wiFfYdH8Gipec8Eeeu0xXdbba9frFj0=OqFfea0dXdd9vqai=hGuQ8kuc9pgc9s8qqaq=dirpe0xb9q8qiLsFr0=vr0=vr0dc8meaabaqaciaacaGaaeqabaqabeGadaaakeaaiiGacqWF1oqzcqGH9aqpdaWcaaqaaiabd6gaUnaaCaaaleqabaGaeGOmaidaaOGaeiikaGIaeGymaeJaeyOeI0Iaem4CamNaeiykaKYaaWbaaSqabeaacqaIYaGmcqGGOaakcqWGUbGBcqGHsislcqaIXaqmcqGGPaqkaaGccqGHsislcqWGUbGBcqGGOaakcqWGUbGBcqGHsislcqaIXaqmcqGGPaqkcqGGOaakcqaIXaqmcqGHsislcqaIYaGmcqWGZbWCcqGGPaqkdaqhaaWcbaGaey4kaScabaGaemOBa4MaeyOeI0IaeGymaedaaaGcbaGagiyBa0MaeiyyaeMaeiiEaGNaeiikaGIaeGymaeJaeiilaWIaemyBa0MaeiykaKcaaaaa@57B5@

*and x*_+ _= max(*x*, 0).

**Corollary 1 ***In the setting of Propositions 1 and 2, the probability that the circle is completely covered is approximately e^-m ^and the absolute error in this approximation is at most ε*.

**Proposition 3 ***Suppose that N arcs, each of length S, are placed uniformly and independently at random on an interval of length 1 (so that the whole of each arc lies on the interval). Then the number of gaps (excluding the gap at each end) has approximately the Poisson distribution with parameter m *= *n*(1 - *s*)^*n*-1^, *where s *= S1−S
 MathType@MTEF@5@5@+=feaafiart1ev1aaatCvAUfKttLearuWrP9MDH5MBPbIqV92AaeXatLxBI9gBaebbnrfifHhDYfgasaacH8akY=wiFfYdH8Gipec8Eeeu0xXdbba9frFj0=OqFfea0dXdd9vqai=hGuQ8kuc9pgc9s8qqaq=dirpe0xb9q8qiLsFr0=vr0=vr0dc8meaabaqaciaacaGaaeqabaqabeGadaaakeaadaWcaaqaaiabdofatbqaaiabigdaXiabgkHiTiabdofatbaaaaa@30F7@*and n *= *N *- 1. *The error in the approximation is just as in Proposition 2, again taking s *= S1−S
 MathType@MTEF@5@5@+=feaafiart1ev1aaatCvAUfKttLearuWrP9MDH5MBPbIqV92AaeXatLxBI9gBaebbnrfifHhDYfgasaacH8akY=wiFfYdH8Gipec8Eeeu0xXdbba9frFj0=OqFfea0dXdd9vqai=hGuQ8kuc9pgc9s8qqaq=dirpe0xb9q8qiLsFr0=vr0=vr0dc8meaabaqaciaacaGaaeqabaqabeGadaaakeaadaWcaaqaaiabdofatbqaaiabigdaXiabgkHiTiabdofatbaaaaa@30F7@*and n *= *N *- 1.

**Corollary 2 ***In the setting of Proposition 3, the probability that no gaps exist except end gaps of length at most d is approximately e^-M^, where*

M=(N−1)(1−2S1−S)N+2(1−S−d1−S)N
 MathType@MTEF@5@5@+=feaafiart1ev1aaatCvAUfKttLearuWrP9MDH5MBPbIqV92AaeXatLxBI9gBaebbnrfifHhDYfgasaacH8akY=wiFfYdH8Gipec8Eeeu0xXdbba9frFj0=OqFfea0dXdd9vqai=hGuQ8kuc9pgc9s8qqaq=dirpe0xb9q8qiLsFr0=vr0=vr0dc8meaabaqaciaacaGaaeqabaqabeGadaaakeaacqWGnbqtcqGH9aqpcqGGOaakcqWGobGtcqGHsislcqaIXaqmcqGGPaqkdaqadaqaamaalaaabaGaeGymaeJaeyOeI0IaeGOmaiJaem4uamfabaGaeGymaeJaeyOeI0Iaem4uamfaaaGaayjkaiaawMcaamaaCaaaleqabaGaemOta4eaaOGaey4kaSIaeGOmaiZaaeWaaeaadaWcaaqaaiabigdaXiabgkHiTiabdofatjabgkHiTiabdsgaKbqaaiabigdaXiabgkHiTiabdofatbaaaiaawIcacaGLPaaadaahaaWcbeqaaiabd6eaobaaaaa@4A9D@

and the absolute error in this approximation is at most

M2−2(1−S−2d1−S)+N−4(N−1)(1−2S−d1−S)+N−(N−1)(N−2)(1−3S1−S)+N
 MathType@MTEF@5@5@+=feaafiart1ev1aaatCvAUfKttLearuWrP9MDH5MBPbIqV92AaeXatLxBI9gBaebbnrfifHhDYfgasaacH8akY=wiFfYdH8Gipec8Eeeu0xXdbba9frFj0=OqFfea0dXdd9vqai=hGuQ8kuc9pgc9s8qqaq=dirpe0xb9q8qiLsFr0=vr0=vr0dc8meaabaqaciaacaGaaeqabaqabeGadaaakeaacqWGnbqtdaahaaWcbeqaaiabikdaYaaakiabgkHiTiabikdaYmaabmaabaWaaSaaaeaacqaIXaqmcqGHsislcqWGtbWucqGHsislcqaIYaGmcqWGKbazaeaacqaIXaqmcqGHsislcqWGtbWuaaaacaGLOaGaayzkaaWaa0baaSqaaiabgUcaRaqaaiabd6eaobaakiabgkHiTiabisda0iabcIcaOiabd6eaojabgkHiTiabigdaXiabcMcaPmaabmaabaWaaSaaaeaacqaIXaqmcqGHsislcqaIYaGmcqWGtbWucqGHsislcqWGKbazaeaacqaIXaqmcqGHsislcqWGtbWuaaaacaGLOaGaayzkaaWaa0baaSqaaiabgUcaRaqaaiabd6eaobaakiabgkHiTiabcIcaOiabd6eaojabgkHiTiabigdaXiabcMcaPiabcIcaOiabd6eaojabgkHiTiabikdaYiabcMcaPmaabmaabaWaaSaaaeaacqaIXaqmcqGHsislcqaIZaWmcqWGtbWuaeaacqaIXaqmcqGHsislcqWGtbWuaaaacaGLOaGaayzkaaWaa0baaSqaaiabgUcaRaqaaiabd6eaobaaaaa@66DC@

## Discussion

We have given bounds on the probability distribution of the number of uncovered gaps when arcs of fixed length are placed randomly on a circle or interval. As discussed in [6], one motivation for such approximations is the issue of computational overflow arising when the exact solution is applied. Typically they involve simple, well-known probability distributions and this aids both computation and further mathematical analysis. Our own motivation in beginning this work was seeing certain quite poor approximations used in practice, both locally and in the literature. For a cautionary example we take *s *= 10^-2 ^and *n *= 750, values which arise when 0.3% of the clones in a metagenomic library consisting of 2.5 × 10^5 ^40-kilobase fosmid inserts are from the genome of interest, and the genome of interest has length 4 megabases. Here ad-hoc approximations using elementary probability theory can indicate a 95% probability that the library completely covers that genome, while the true probability is 66% (neglecting biologically related bias; calculations are to the nearest integer and are not given).

We stress the similarity between our approximations and those already given in the literature. In [2,3,7] simplifying assumptions are made which give a binomial distribution for the number of gaps. Our approximation is a Poisson distribution, and it is well known in probability theory that the binomial distribution converges to the Poisson distribution in certain limits; one such convergence is proved in [8]. Indeed, these different approximations are generally numerically close. The contribution of the present paper is therefore to provide an approximation with error bounds.

We refer the reader to [2] for a simple modification to coverage problems when a certain minimum overlap is required between arcs, and give a worked example in Additional file 2. Note that in [2] the expected number of gaps is calculated approximately; as shown in Additional file 1, the exact value for the expected number of gaps is *m *= *n*(1 - *s*)^*n*-1^. This is the parameter of the Poisson distribution in Proposition 1. Our approximating Poisson distribution therefore has the same expectation as the exact distribution, although its variance differs (the exact variance of the number of gaps is given in Additional file 1).

Our results may be applied to the stopping problem. Suppose we desire *p*% probability that no more than *w *gaps remain at the end of the undirected part of a shotgun sequencing project. By inverting Proposition 1 (see Additional file 2 for details) we obtain an approximate value for the number of clones, and hence coverage depth, required. Here again, our contribution is not the estimate but rather the lower bound given by Proposition 2 for the probability that no more than *w *gaps remain (neglecting biologically related bias). Other solutions to the stopping problem have been proposed: see for example [6] and [3] for alternative strategies and further discussion of the stopping problem.

**Figure 1:**
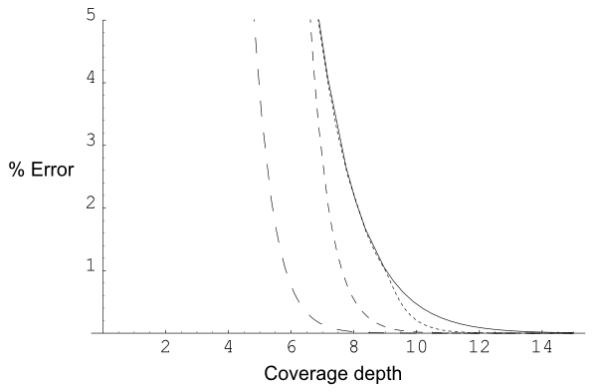
**Error bounds**. Error bound for the approximate distribution of the number of gaps when a circle is covered by random arcs. The error bound (given as an absolute error, measured in percentage points) is plotted against coverage depth, for arc lengths *s *= 10^-1^, 10^-2^, 10^-3 ^(progressively smaller dashes) and 10^-7 ^(solid).

The practical relevance of our approximations clearly depends on the size of the error bound. Figure 1 plots the error bound against coverage depth for arc lengths *s *= 10^-1^, 10^-2^, 10^-3 ^and 10^-7 ^(the curves for *s *= 10^-4^, 10^-5 ^and 10^-6 ^are almost indistinguishable from the *s *= 10^-7 ^curve at this scale). We emphasise that *s *is the *relative *arc length – so for genomic applications, *s *is the actual arc length divided by the length of the genome. These arc lengths are intended to represent the full range of typical genomic projects: for example the smallest, *s *= 10^-7^, would correspond to covering the largest known eukaryote genome, the amoeba *Chaos chaos *[9], with 400 kilobase bacterial artificial chromosome (BAC) inserts. A lookup table of relative arc lengths for recent shotgun sequencing projects is given in [4]. It can be seen that for these relative arc lengths, error bounds on the order of 0.1% are achieved at 10× coverage. Further discussion of Figure 1 is given in Additional file 1; it should also be remarked that the error bound at 5× coverage is considerably larger. For the particular experimental parameters relevant to the user, the spreadsheet in Additional file 3 may be used to obtain values for the approximation and error bounds.

The validity of coverage problems in general for genomic applications depends of course on the extent to which they capture the actual problem. For example, a given genome might contain one gene which is toxic to the *E. coli *host in a BAC library. Since library fragments will not contain this gene, the corresponding coverage problem is on the interval rather than the circle. With *a priori *knowledge of an unclonable region we may therefore apply Proposition 3 rather than Proposition 1; without such information we may choose either to neglect this effect or to model it (for example using Propositions 1 and 3 and conditional probability). Pathological cases certainly exist, for example the highly repetitive maize genome for which as many as 80% of arc positions may not be cloned [7]. The approach should therefore be chosen using the best available information. Certain other biases are not so dependent on the particular target genome, for example the inevitable small variations in clone length and position bias. These have been discussed in [3,4,10] using empirical data and simulations and the consensus is that they may be neglected. The interested mathematical reader may check that by first conditioning on the fragment lengths, our method gives a lower bound for *P*(*W *≤ *w*) in Proposition 2 when the arc lengths are random, although we do not pursue this.

Another modelling issue arises in metagenomics, which is the culture-independent study of a mixed community of genomes. In a metagenomic library the number of clones *n *from a genome in the community is random, having a binomial distribution (in the absence of bias). If the composition of the community is known then, from the central limit theorem of probability, *n *is well approximated by its average and this value may be used in Proposition 1 (see Additional file 2). Further, since the distribution of *n *is concentrated at a few values around its mean, it is typically computationally inexpensive to obtain satisfactory bounds for the distribution of the number of gaps by an application of conditional probability.

## Authors' contributions

JRM brought the problem to the attention of the other authors and posed the worked examples [see Additional file 2]. AM wrote the mathematical proofs [see Additional file 1]. JM supervised the study and wrote the remainder. All authors read and approved the final manuscript.

## Supplementary Material

Additional File 1 **Methods**. Mathematical proofs. Click here for file

Additional File 2 **Practical application to genomics and metagenomics**. Worked example applications in genomics and metagenomics. Click here for file

Additional File 3 **Calculator**. A calculator implementing the formulas in Propositions 1 and 2. Click here for file
