# Supplementary material for: Bounds on the distribution of the number of gaps when circles and lines are covered by fragments: Theory and practical application to genomic and metagenomic projects
Source: BMC Bioinformatics. 2007 Mar 2;8:70. doi: 10.1186/1471-2105-8-70 (PMC1821341; doi:10.1186/1471-2105-8-70)
Supplement: Additional File 1 — Methods. Mathematical proofs. [file 1471-2105-8-70-S1.pdf]

Additional file 1: Methods

Proof of Propositions 1 and 2

Consider a circle with circumference of length 1. Place n particles $X_1, \ldots, X_n$ uniformly and independently at random on the circle. Choose one of the particles and label it $X_{n,1}$. We may imagine that the circle is cut at this point, and opened out into a line segment of length 1. We then label one end of the line segment as the point 0, and the other end as the point 1. Let $X_{n,2}, \ldots, X_{n,n}$ denote the positions on the line segment of the remaining $n-1$ particles, arranged in increasing order: $0 = X_{n,1} \leq X_{n,2} \leq \cdots \leq X_{n,n} \leq 1$. Let L_k be the distances between neighbouring points,

$$L_k = \begin{cases} X_{n,k+1} - X_{n,k} & \text{if } 1 \leq k \leq n-1 \\ 1 - X_{n,n} & \text{if } k = n. \end{cases}$$

This is illustrated in Figure 2.

We wish to approximate the probability that $L_k \leq s_k$ for all except w of the k's, where the $s_1, \ldots, s_n$ are given real numbers between 0 and 1: that is,

$$\mathbb{P}[W(s_1, \ldots, s_n) \leq w]$$

where $W(t_1, \ldots, t_n) = \sum_{k=1}^{n} \chi_k(t_k)$ and the function $\chi_i(s)$ takes the value 1 if $L_i > s$ and 0 otherwise. We will apply the Chen-Stein method, which provides an approximation provided we can calculate the first two moments of W and establish certain properties of the χ_i.

Theorem 1 (from Lindvall, 2002) *Let $\{Y_i\}_{i=1}^{n}$ be Bernoulli random variables on a probability space $(\Omega, \mathcal{F}, \mathbb{P})$, and let $W = \sum_{i=1}^{n} Y_i$. For each $1 \leq k \leq n$ let U_k and V_k be random variables with $U_k =^d W$, and $1 + V_k$ having distribution*

$$\mathbb{P}[W \in \cdot | Y_k = 1]$$

($=^d$ denotes equality in distribution). Then

$$\sup_{A \subseteq \mathbb{Z}_+} |\mathbb{P}[W \in A] - p_{\mathbb{E}[W]}[A]| \leq (1 \wedge \mathbb{E}[W]^{-1}) \sum_{k=1}^{n} \mathbb{P}[Y_k = 1] \mathbb{E}[|U_k - V_k|],$$

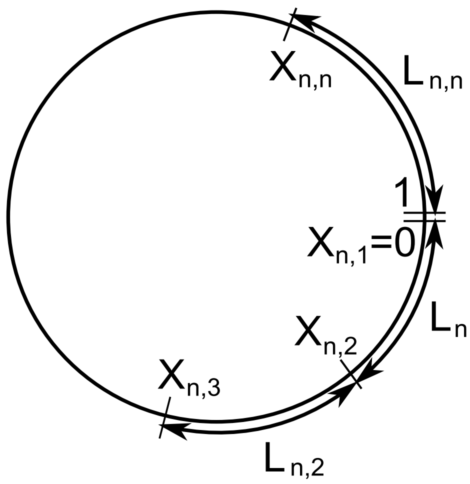

Figure 2: Arc and particle positions on the circle.

where p_λ represents the Poisson distribution with parameter λ. In particular when $U_k \geq V_k$ for all k we have

$$\sup_{A\subseteq\mathbb{Z}_+} |\mathbb{P}[W \in A] - p_{\mathbb{E}[W]}[A]| \leq (1 \wedge E[W]^{-1})(\mathbb{E}[W] - Var[W]) \qquad (1)$$

where $Var[W]$ is the variance of W.

We first verify the conditions of Theorem 1. For each i set $Y_i = \chi_i(s_i)$, and for each k put $U_k = W$ and $V_k = \sum_{i\neq k} \chi_i(s_i/(1-s_k))$. Clearly $U_k \geq V_k$ for all k; to see that the distributions $\mathbb{P}[1 + V_k \in \cdot]$ and $\mathbb{P}[W \in \cdot | Y_k = 1]$ are equal, we have

$$\mathbb{P}[W = m|\chi_k(s_k) = 1] = \frac{\mathbb{P}\left[\sum_{i\neq k} \chi_i(s_i) = m-1, \chi_k(s_k) = 1\right]}{\mathbb{P}[\chi_k(s_k) = 1]}.$$

The event in the numerator can only occur if both $\chi_k(s_k) = 1$ and there exist $i_1 < \ldots < i_{m-1} \in \{1, \ldots, n\} \setminus \{k\}$ such that $\chi_{i_j}(s_{i_j}) = 1$ for $1 \leq j \leq m-1$, and $\chi_l(s_l) = 0$ otherwise. Applying the definition of χ_i we have

$$\begin{aligned} &\mathbb{P}[W = m|\chi_k(s_k) = 1] \qquad (2)\\ &= \sum_{\substack{i_1<\ldots<i_{m-1}\in\\ \{1,\ldots,n\}\setminus\{k\}}} \frac{\mathbb{P}[L_{i_1} > s_{i_1}, \ldots, L_{i_{m-1}} > s_{i_{m-1}}, L_k > s_k, L_l \leq s_l \text{ otherwise}]}{\mathbb{P}[L_k > s_k]}\end{aligned}$$

Now

$$\mathbb{P}[1 + V_k = m] = \mathbb{P}\left[\sum_{i\neq k} \chi_i\left(\frac{s_i}{1-s_k}\right) = m-1\right]$$

and similarly the above event can only occur if there exist $i_1 < \ldots < i_{m-1} \in \{1,\ldots,n\} \setminus \{k\}$ with $\chi_{i_j}(s_{i_j}/(1-s_k)) = 1$ for $1 \le j \le m-1$ and $\chi_l(s_l/(1-s_k)) = 0$ otherwise:

$$\mathbb{P}[1+V_k = m] = \sum_{\substack{i_1<\ldots<i_{m-1}\in \\ \{1,\ldots,n\}\setminus\{k\}}} \mathbb{P}\left[L_{i_1} > \frac{s_{i_1}}{1-s_k},\ \ldots, L_{i_{m-1}} > \frac{s_{i_{m-1}}}{1-s_k},\right.$$
$$\left. L_k \in \mathbb{R}, L_l \le \frac{s_l}{1-s_k} \text{ otherwise}\right] \quad (3)$$

The equality of $\mathbb{P}[W = m|Y_k = 1]$ and $\mathbb{P}[1+V_k = m]$ then follows by applying the inclusion-exclusion formula, Lemma 1 to equations (2) and (3) as follows.

$$\begin{aligned}
&\mathbb{P}\left[L_{i_1} > \frac{s_{i_1}}{1-s_k},\ldots, L_{i_{m-1}} > \frac{s_{i_{m-1}}}{1-s_k}, L_k \in \mathbb{R}, L_l \le \frac{s_l}{1-s_k} \text{ otherwise}\right] \\
&= \sum_{j=1}^{n-m} \sum_{a_1<\cdots<a_j} (-1)^j \mathbb{P}\left[L_l > \frac{s_l}{1-s_k} \text{ for } l = i_1,\ldots,i_{m-1},\ a_1,\ldots,a_j\right] \\
&= \sum_{j=1}^{n-m} \sum_{a_1<\cdots<a_j} (-1)^j \left(1 - \left(\frac{\sum_{l=1}^{m-1} s_{i_l} + \sum_{l=1}^{j} s_{a_l}}{1-s_k}\right)\right)_+^{n-1} \\
&= \sum_{j=1}^{n-m} \sum_{a_1<\cdots<a_j} (-1)^j \frac{(1-s_k - \sum_{l=1}^{m-1} s_{i_l} - \sum_{l=1}^{j} s_{a_l})_+^{n-1}}{(1-s_k)^{n-1}} \\
&= \sum_{j=1}^{n-m} \sum_{a_1<\cdots<a_j} (-1)^j \frac{\mathbb{P}[L_l > s_l \text{ for } l = i_1,\ldots,i_{m-1},k,a_1,\ldots,a_j]}{\mathbb{P}[L_k > s]} \\
&= \frac{\mathbb{P}[L_{i_1} > s_{i_1},\ldots,L_{i_{m-1}} > s_{i_{m-1}}, L_k > s_k, L_l \le s_l \text{ otherwise}]}{\mathbb{P}[L_k > s]}.
\end{aligned}$$

where it is understood that in the sums, no a_l is equal to k or to any i_l.

To calculate the required moments of W we use the following result:

Lemma 1 (Stevens, 1939) *For any subset $I \subseteq \{1,\ldots,n\}$ and any $t_k \in [0,1]$ for all $k \in I$ we have*

$$\mathbb{P}[L_k > t_k \textit{ for all } k \in I] = \left(1 - \sum_{k\in I} t_k\right)_+^{n-1},$$

Then by the definition of W and Lemma 1

$$\mathbb{E}[W(s_1,\ldots,s_n)] = \sum_{k=1}^{n} (1-s_k)^{n-1}$$

The variance of W is

$$\begin{aligned}\mathrm{Var}[W(s_1, \quad \dots \quad, s_n)] &= \mathbb{E}[W(s_1,\dots,s_n)^2] - \mathbb{E}[W(s_1,\dots,s_n)]^2 \\ &= \sum_{k=1}^{n}(1-s_k)^{n-1} + \sum_{l\neq k}(1-s_l-s_k)_+^{n-1} - \mathbb{E}[W(s_1,\dots,s_n)]^2\end{aligned}$$

Setting all s_i equal to s gives Propositions 1 and 2.

Proof of Proposition 3

This follows from the above proof after the following observations:

1. In the above proof, placing N random points on an interval is equivalent to placing $N+1$ random points on a circle. Neglecting the end gaps is then equivalent to considering $W' = \sum_{i=2}^{N} Y_i$. Calculation of the mean and variance of W' gives the error bound in Proposition 2 when $n = N-1$.

2. If N arcs of length S are placed uniformly at random on an interval of length 1 so that each lies wholly on the interval, then their left endpoints are uniformly distributed on the interval $(0, 1-S)$. By scaling, we may therefore use the above proof with $n = N+1$ and $s = S/(1-S)$. Also by scaling, the requirement that no uncovered gaps exist except end gaps of length at most d is equivalent to taking $s_1 = s_{N+1} = \frac{d}{1-S}$, $s_i = \frac{S}{1-S}$ otherwise.

Discussion of Figure 1

It should not be inferred from Figure 1 that the error bound curves converge as $s \to 0$. Indeed, Huillet (2003) proves that for a fixed coverage depth, the number of gaps almost surely tends to infinity as $s \to 0$.
