# Supplementary material for: Bounds on the distribution of the number of gaps when circles and lines are covered by fragments: Theory and practical application to genomic and metagenomic projects
Source: BMC Bioinformatics. 2007 Mar 2;8:70. doi: 10.1186/1471-2105-8-70 (PMC1821341; doi:10.1186/1471-2105-8-70)
Supplement: Additional File 2 — Practical application to genomics and metagenomics. Worked example applications in genomics and metagenomics. [file 1471-2105-8-70-S2.pdf]

Additional file 2: Practical application to genomics and metagenomics

We give here examples of questions concerning genomic and metagenomic libraries which may be answered using our results. Numerical examples are given, using values which are typical in experimental work local to the authors where possible. We assume that a library consisting of clones of equal length is constructed from a community of circular genomes and we neglect biologically related biases. One genome is of particular interest and we assume that it comprises a known proportion of the community. Introduce the following symbols:

$$
\begin{aligned}
X &= \text{genome of interest} \\
G &= \text{length of the genome of interest, in megabases (Mb)} \\
k &= \text{number of clones in the library} \\
l &= \text{length of each clone in Mb} \\
a &= \text{proportion of } X \text{ in the community (by genetic material)} \\
n &= \text{random variable representing the number of library clones coming from } X \\
s &= \frac{l}{G}
\end{aligned}
$$

1. *What is the probability that genome X has*
   A. at least one fragment in the metagenomic library,
   B. its entire genome in the metagenomic library?

The answer to part A is $1-(1-a)^k$ (and this may be recognised as the formula of Clarke and Carbon (1976), who consider a related problem). As outlined in the Discussion, Propositions 1 and 2 provide both an approximation and error bounds for part B. A simplifying assumption is that the number of clones in the library coming from species X is ak, and Proposition 1 may then be applied. This simplifying assumption can be avoided with an application of conditional probability, and error bounds can again be obtained; however this is not pursued here.

As a worked example take $G = 4, k = 250000, l = 0.04, a = 0.0036$. Then the answer to part A is

$$1-(1-0.0036)^{250000} = 1.000$$

In other words, if X consititutes 0.36% of the community by genetic material then the probability that at least one fragment in the metagenomic library is from X is 100.0% (to 1 decimal place). For part B we apply Proposition 1. We have $ak = 900$ and $s = \frac{l}{G} = 0.01$. We desire the probability that the genome is completely covered by fragments - that is, that there are 0 gaps. From elementary theory, the probability that a Poisson random variable with parameter m takes the value 0 is e^{-m}. Since $m = n(1-s)^{n-1} = 0.1072$, the approximate answer is therefore

$$e^{-0.1072} = 0.8983 = 89.83\%$$

For the error bound we apply Proposition 2 (with $w = 0$). The error bound is $\epsilon = 0.0011 = 0.11\%$. The true probability therefore lies between 89.72% and 89.94%. An extra decimal place has been given here to demonstrate the similarity of our approximation with those already given in the literature: the approximation of Roach (1995) and the asymptotic approximation of Wendl and Waterston (2002) are $(1-(1-s)^n)^{n-1} = 89.94\%$ and $(1-e^{-s(n-1)})^n = 89.39\%$ respectively (they do not provide error bounds).

2. In the case of a genomic library, what is an approximate probability with error bounds that the entire genome is in the library, with an overlap of at least 100 bases between fragments?

To answer this question, simply apply Propositions 1 and 2 with the fragment length l reduced by 0.0001 Mb (100 bases) to account for the required overlap.

3. For a metagenomic library, what percentage of the community must a given genome constitute in order to have a probability P that

A. at least one fragment from that genome is in the library,

B. the entire genome is in the library?

Rearranging the solution to question 1A, we find that the answer to part A is

$$100\left(1-(1-P)^{1/k}\right)$$

For example, if $k = 250000$ and we require a 99% confidence level then we take $P = 0.99$ and

$$100\left(1-(1-0.99)^{1/250000}\right) = 0.0018$$

so the genome must constitute at least 0.0018% of the community by genetic material.

For part B, Corollary 1 gives the equation $P = e^{-n(1-s)^{n-1}}$ which cannot easily be rearranged, so a numerical solver may be used to find the required percentage value. A simple spreadsheet for the calculation is available in Additional file 3.

4. How many clones should be in a metagenomic library in order to have a probability P that

A. at least one fragment from X is in the library,

B. the whole of genome X is in the library?

For part A we may again rearrange the solution to question 1A, giving

$$k = \frac{\log(1-P)}{\log(1-a)} \tag{1}$$

As an example, if $a = 0.0004$ and $P = 0.99$ then the required library size is 11511 fragments. For part B we may again use a numerical solver (see answer to question 3).

5. In the undirected part of a shotgun sequencing project, how many fragments are required to give an approximate 95% probability that no more than 2 gaps remain at the end?

Here we must invert Proposition 1 to find the required value of n, and a numerical solver such as additional file 3 may be used. As an example take $G = 10, l = 0.02$: then $s = 0.002$ and solving numerically gives $n = 4278$. Putting this value of n back into Proposition 2, we see that the error bound is 1.2%.

6. Suppose that X has an unclonable region. Given that the clonable region is an interval I of length 2 Mb, what is the probability that I is completely covered except for the end gaps, which are of length at most 0.1 Mb, by 50 fragments of length 0.2 Mb?

We apply Corollary 2. We have $S = 0.1$ and $d = 0.05$, so the probability is approximately 77.8% and the error bound is 2.8%.

7. What is the effect of changing the fragment length in the above calculations?

The answers to questions 1A, 3A and 4A are unchanged. The answers to all other questions given above will change; it is clear that longer fragments give a higher probability that a particular complete genome is represented in the metagenomic library, and *vice versa*. To obtain the values we must repeat the calculations using the value of s corresponding to the new fragment length.
